# Proteome Analysis of Outer Membrane Vesicles From a Highly Virulent Strain of *Haemophilus parasuis*

**DOI:** 10.3389/fvets.2021.756764

**Published:** 2021-11-26

**Authors:** Kunli Zhang, Pinpin Chu, Shuai Song, Dongxia Yang, Zhibiao Bian, Yan Li, Hongchao Gou, Zhiyong Jiang, Rujian Cai, Chunling Li

**Affiliations:** ^1^Institute of Animal Health, Guangdong Academy of Agricultural Sciences, Key Laboratory of Livestock Disease Prevention of Guangdong Province, Scientific Observation and Experiment Station of Veterinary Drugs and Diagnostic Techniques of Guangdong Province, Ministry of Agriculture and Rural Affairs, Guangzhou, China; ^2^Maoming Branch, Guangdong Laboratory for Lingnan Modern Agriculture, Guangdong, China

**Keywords:** *Haemophilus parasuis*, outer membrane vesicles, purification, proteome analysis, inflammatory response

## Abstract

*Haemophilus parasuis* has emerged as an important bacterial pathogen in pig husbandry, as *H. parasuis* can coinfect pigs with a variety of pathogenic microorganisms and further cause an aggravation of the disease. It is crucial to investigate its pathogenetic mechanism. Gram-negative bacteria naturally secrete outer membrane vesicles (OMVs), and their potent virulence factors play prominent roles that affect the interaction between bacteria and host. Still, the pathogenesis that is associated with the bacterial OMVs has not been well-elucidated. In this study, we investigated the secretion of OMVs from a clinical *H. parasuis* isolate strain (H45). In addition, we further analyzed the characterization, the comprehensive proteome, and the virulence potential of OMVs. Our data demonstrated that *H. parasuis* could secrete OMVs into the extracellular milieu during infection. Using liquid chromatography with tandem mass spectrometry (MS/MS) identification and bio-information analysis, we identified 588 different proteins associated with OMVs. Also, we also analyzed the subcellular location and biological function of those proteins. These proteins are mainly involved in immune and iron metabolism. Moreover, we confirmed the pathogenicity of *H. parasuis* OMVs by observing a strong inflammatory response in J774A.1 and porcine alveolar macrophages. Taken together, our findings suggested that OMVs from *H. parasuis* were involved in the pathogenesis of this bacterium during infection.

## Introduction

*Haemophilus parasuis* is a Gram-negative bacterium of the Pasteurellaceae family. *Haemophilus parasuis* is an early colonizer of the upper respiratory tract and also is a part of the normal microbiota of healthy pigs (1). *Haemophilus parasuis* is the causative agent of Glässer's disease, which is characterized by fibrinous polyserositis, polyarthritis, meningitis, and arthritis and causes significant economic losses. Glässer's disease prevention and control continue to be challenging, as the pathogenicity of *H. parasuis* is not fully characterized. Fifteen standard *H. parasuis* serovars have been recognized in addition to non-typeable isolates. Among the standard serovars, 1, 5, 10, 12, 13, and 14 are considered highly virulent, 2, 4, 15, and 8 are mildly virulent, and serovars 3, 6, 7, 9, and 11 are considered avirulent (2, 3). Serovars 4 and 5 are the most prevalent in China, followed by serovars 7, 12, 13, and 14 (4–6). Virulence factors play a primary role during the bacterial pathogenic process. So far, various virulence factors of *H. parasuis* have been identified, including 6-phosphogluconate dehydrogenase, lipooligosaccharide, cytolethal distending toxin protein (CDT, CdtA, CdtB, and CdtC), immunoglobulin A protease, capsule, porin family proteins (OmpP2 and OmpP5), monomeric autotransporters (bmaA), virulence-associated trimeric autotransporters (VtaA2, VtaA3, vtaA8, and vtaA9), and GalU and GalE (7–13). These virulence factors are involved in different pathogenesis processes, including invasion of the host, evasion of host's defenses, and the assistance of bacterial multiplication.

It has been found that most Gram-negative bacteria can secrete outer membrane vesicles (OMVs) during both *in vitro* growth and *in vivo* infection (14). Studies show that OMVs are 20–250 nm in diameter, containing lipopolysaccharide (LPS), outer membrane proteins (OMPs), outer membrane lipids, periplasmic proteins (OPs), cytoplasmic proteins (CPs), and DNA or RNA (15). Several kinds of pathogenic Gram-negative bacteria secrete OMVs, including *Escherichia coli* (16), *Neisseria meningitidis* (17), *Shigella flexneri* (18), *Helicobacter pylori* (19), and *Pseudomonas aeruginosa* (20). OMVs contain diverse virulence factors of pathogenic bacteria, such as heat-labile toxin and cytolysin A of *E. coli*, b-lactamase, hemolytic phospholipase C of *P. aeruginosa*, and VacA of *H. pylori*, and their roles in bacterial pathogenesis have been relatively well-characterized (21). OMVs also play important roles in microbial community interactions, including quorum sensing, biofilm formation, nutrient acquisition, antibiotic resistance, and competition against other microbes. For example, the hydrophobic quorum senses molecules *Pseudomonas* quinolone signal of *P. aeruginosa*, C16-HSL of *Paracoccus denitrificans*, and CAI-1 of *Vibrio harveyi* are packaged into OMVs, which allows for stabilization, concentration, and dispersal through the environment to mediate the generation of virulence factors, iron acquisition, and host immune responses (22–24). In the case of *Haemophilus influenzae*, higher OMV levels correlated with increased serum resistance (25). Recent research found that outer membrane vesiculation facilitates surface exchange and *in vivo* adaptation of *vibrio cholera* (26). However, the secretion of OMVs and their contribution to pathogenesis have been rarely reported in *H. parasuis*. In the present study, we have purified the OMVs from a clinical *H. parasuis* and analyzed the proteome of the *H. parasuis*-derived OMVs. We demonstrated that *H. parasuis* could secrete OMVs during *in vitro* growth. The liquid chromatography with MS/MS analysis showed that several virulence-associated proteins and immune modulators from *H. parasuis*-derived OMVs were identified. Moreover, we found that OMVs of *H. parasuis* could induce interleukin (IL)-1β production in mouse mononuclear macrophages (J774A.1) and porcine alveolar macrophages (PAMs). These results suggest that OMVs can be vehicles for the transport of effector molecules into host cells.

## Results

### *Haemophilus parasuis* Secretes Outer Membrane Vesicles During *in vitro* Growth

**Figure 1:**
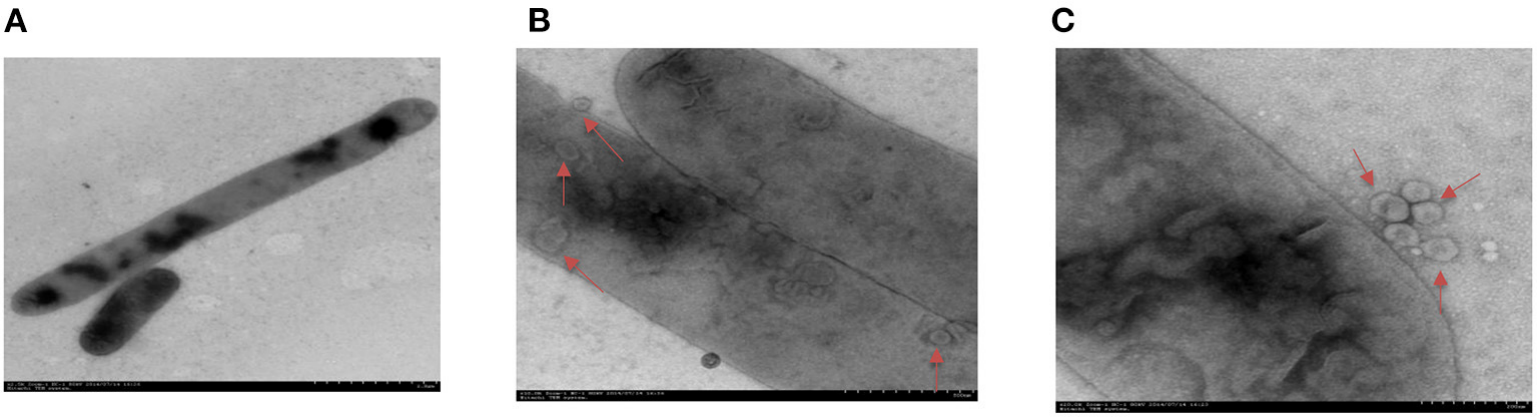
Transmission electron micrographs image of *H. parasuis* and nascent OMVs. **(A)** Image of *H. parasuis* H45 strain which stained with uranyl acetate. **(B)** Transmission electron micrographs image of OMVs (red arrow) located at intracellular of *H. parasuis* H45 strain. **(C)** Transmission electron micrographs image of OMVs (red arrow) located at extracellular of *H. parasuis* H45 strain.

To investigate whether *H. parasuis* can secrete OMVs during *in vitro* growth, a clinical isolate of *H. parasuis* H45 was grown in tryptic soy broth (TSB) and observed with electron microscopy. The H45 strain had the typical morphology of HPS, which has various forms, from a single short bacillus to a long rod (Figure 1A). Further observation revealed that some vesicle-like structures were found in and around the bacterium (Figures 1B,C). As displayed in Figure 1C, the spherical vesicles budded from bacterial outer membranes. These results suggested that *H. parasuis* H45 could secrete OMVs during *in vitro* growth.

### Purification and Characterization of H45-derived Outer Membrane Vesicles

**Figure 2:**
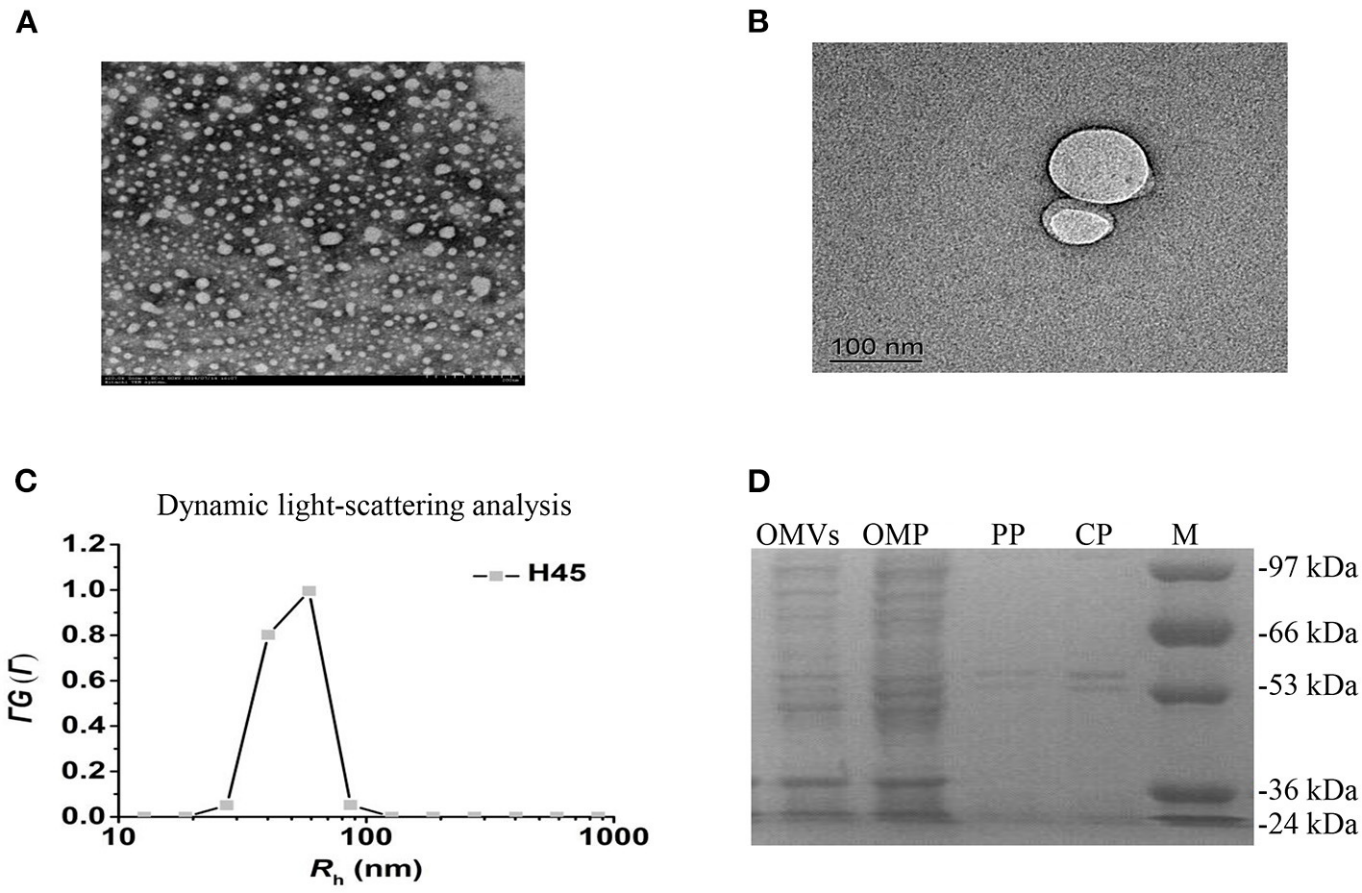
Characteristics of OMVs isolated from *H. parasuis* H45 strain. **(A)** Negatively stained preparation of isolated *H. parasuis* OMVs showing size of OMVs. Scale bar: 200 nm. **(B)** Transmission electron microscopy image of generation of one OMV from H45. Scale bar: 100 nm. **(C)** CONTIN analysis of dynamic light scattering data. **(D)** Comparison of protein components in different parts of HPS by SDS-PAGE. OMV, Outer membrane vesicles; OMP, Outer membrane protein; PP, Periplasmic protein; CP, Cytoplasmic protein.

The cell-free OMV fractions isolated from *H. parasuis* broth cultures were repurified by density gradient ultracentrifugation. The electron microscopy results showed that OMVs' fraction exhibited bilayered spherical vesicles (Figures 2A,B). The vesicle diameters ranged from 20 to 160 nm, with an average 50–70 nm diameter. Dynamic light scattering resulted that the Rh of the vesicles is ~69.8 nm; the size of purified OMVs ranged in diameter from ~50 to 100 nm, which was consistent with other reports (Figure 2C). To further determine whether the vesicles carried bacterial proteins, OMPs, OPs, and CPs were subjected to sodium dodecyl sulfate-polyacrylamide gel electrophoresis (SDS-PAGE) along with OMV fraction (Figure 2D). High similarity of protein bands among OMP, OP, CP, and the OMV fraction was observed, suggesting that the vesicles were mainly responsible for carrying the extracellular proteome of *Haemophilus parasuis*.

### Proteome Analysis of *Haemophilus parasuis*-Derived Outer Membrane Vesicles

**Figure 3:**
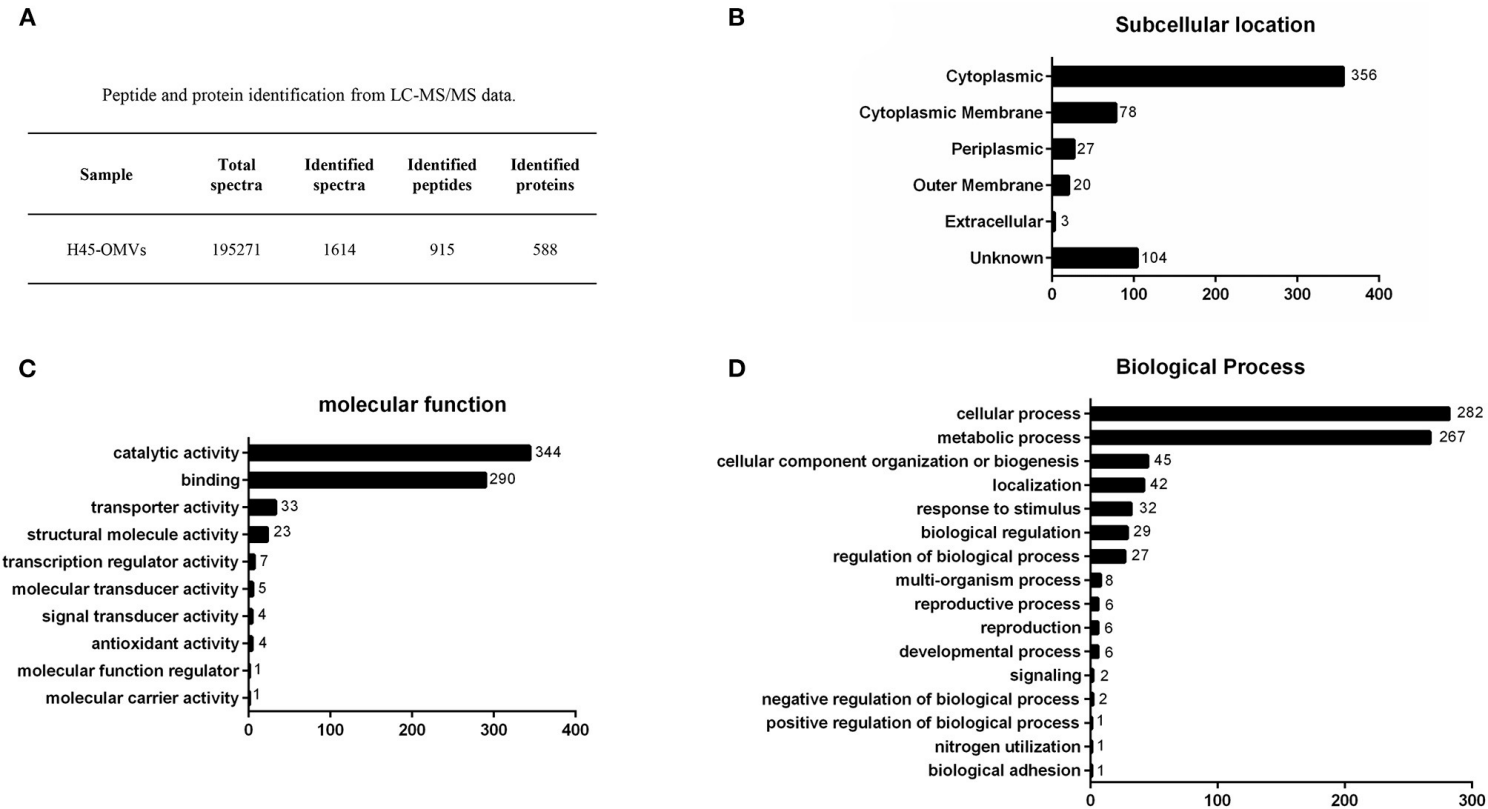
Overview of proteome identification of *H45*-derived OMVs. **(A)** Peptide and protein of OMVs from liquid chromatography with tandem mass spectrometry data. **(B–D)** All identified proteins of H45-OMVs were analyzed by GO functional annotation (http://www.geneontology.org.), subcellular location **(B)**, molecular function **(C)**, and biological process of genes **(D)**.

To identify proteins associated with *H. parasuis*-derived OMVs, proteome analysis was performed by liquid chromatography with MS/MS. Firstly, the purified OMVs were digested with trypsin enzyme to obtain the freeze-dried peptide liquid without salt. Then, the dried peptide samples were separated by Shimadzu LC-20AD model nanoliter liquid chromatography. The liquid phase chromatography-separated peptides were detected by electrospray ionization (ESI) MS/MS (Triple TOF 5600). Subsequently, aligning the experimental MS/MS data with theoretical MS/MS data from UniProt and National Center for Biotechnology Information database is used to identify the proteins of OMVs. In all, 195,271 spectra were obtained. After identification by the search engine, 1,614 spectra were matched; 915 peptides and 588 proteins were identified in *H. parasuis* OMVs (Figure 3A). The mass spectrometry proteomics data have been deposited to the ProteomeXchange Consortium (http://proteomecentral.proteomexchange.org) via the iProX partner repository with the dataset identifier PXD029451. The identified proteins were classified into six groups according to the subcellular location of the proteins in the bacteria: cytoplasmic (*n* = 356), cytoplasmic membrane (*n* = 78), periplasmic (*n* = 27), outer membrane (*n* = 20), extracellular (*n* = 3), and unknown (*n* = 104) (Figure 3B). OMVs are enriched in membrane proteins (*n* = 98), including CPs and OMPs. In contrast, 5% of the total bacterial proteins are OPs. Those subcellular location results indicated that the OMVs were successfully purified from *Haemophilus parasuis*. Notably, proteomic results indicated that a large number of CPs were highly enriched in *H. parasuis* OMVs. Furthermore, we carried out a Gene Ontology (GO) functional annotation analysis for all identified proteins. The molecular function and biological processes genes are shown in Figures 3C,D. Similar to other reports, the *H. parasuis* OMVs were rich in proteins that have catalytic activity (*n* = 344) and binding function (*n* = 290) (Figure 3C). The classification of biological processes showed that *H. parasuis*-derived OMVs also contain numbers of proteins associated with cellular processes (*n* = 282) and metabolic processes (*n* = 257) (Figure 3D). These results suggested that OMVs play important roles in the pathogenesis of *H. parasuis* during infection.

### Outer Membrane Vesicles Carry Immune Modulators and Virulence-associated Proteins

OMVs are considered to represent a specialized bacterial secretion pathway. Several studies showed that OMVs could deliver bacterial bioactive proteins, toxins, and virulence factors (15). According to the proteome result of OMVs, we found that *H. parasuis* (H45)-derived OMVs are rich in immune modulators and virulence-associated proteins. According to the protein mass score, the detected proteins related to immune modulators and virulence-associated proteins are listed in Table 1. Lots of immunogenic proteins are identified, such as ompP2 (ACL31860.1), oppA (ACL32119.1), ompD15 (ACL31912.1), palA (ACL31779.1), AfuA (ACL31862.1), LppC (ACL32393.1), espP2 (ACL32961.1), and so on. These results indicated that OMV might induce a degree of protective immunity and could be selected as a new type subunit vaccine to prevent *H. parasuis* infection. Iron is an essential nutrient for almost all bacteria. To acquire enough free iron from the host, pathogenic bacteria have developed different mechanisms to obtain iron from the host (siderophores, hemophores, or host-molecule-binding proteins), which are involved in the expression of surface-exposed proteins (27). The iron metabolism-related proteins yfeA (ACL32714.1), dsbC (ACL31879.1), hxuA (ACL33616.1), fbpA (ACL32742.1), and HAPS_0679 (ACL32327.1) were found in OMV proteome data. In addition, several virulence-associated proteins also were identified: they are vtaA9 (ADZ54070.1), tbpA (ACL33656.1), pgi (ACL32784.1), manB (ACL32480.1), ompP5 (ACL33726.1), TolC (ACL33604.1), and yfeA (ACL32714.1). Serum resistance is potential pathogenesis of *H. parasuis*, which is associated with systemic diseases in swine. Several serum-resistance associated proteins of *H45*-derived OMVs were identified, including ctdA (ACL32011.1), ctdB (ACL32010.1), ctdC (ACL32009.1), lsgB (ACL31747.1), galE (ACL32657.1), HAPS_1019 (ACL32638.1), and oppA (ACL32119.1). Among them, ctdA, ctdB, ctdC are considered to contribute to cell cycle arrest, apoptosis, adherence, and invasion. *Haemophilus parasuis* (H45)-derived OMVs can carry such numbers of proteins that associated with virulence and/or pathogenic mechanism, which suggested OMVs might have an important role in *H. parasuis* infection.

**Table 1:** Major proteins identified from OMVs of *H. parasuis* H45 strain.

**Protein_ID**	**Protein_Mass**	**Gene Name**	**Description**	**Molecular_Function**
ACL31860.1	39063.77611	ompP2	Outer membrane protein P2 precursor	Immunogenic proteins; serum resistance
ACL32119.1	58257.91922	oppA	Putative oligopeptide transporter, periplasmic-binding protein	Immunogenic proteins
ACL31912.1	66191.56571	ompD15	Surface antigen (D15)	Immunogenic proteins
ACL31779.1	16378.14523	palA	18K peptidoglycan-associated outer membrane lipoprotein	Immunogenic proteins
ACL31862.1	38318.96807	AfuA	Iron (Fe3+) ABC superfamily ATP binding cassette transporter	Immunogenic proteins
ACL32393.1	63954.67071	LppC	Lipoprotein C/GS60 antigen	Immunogenic proteins
ACL32961.1	85477.42837	espP2	Putative extracellular serine protease (autotransporter)	Immunogenic proteins
ACL32476.1	46061.09843	oapA	Opacity associated protein A	Immunogenic proteins
ACL31707.1	36265.64772	gapA	Glyceraldehyde-3-phosphate dehydrogenase	Immunogenic proteins
ACL32554.1	29940.87234	HAPS_0926	DNA uptake lipoprotein, TPR repeat-containing protein	Immunogenic proteins
ACL33700.1	28021.30356	VacJ	VacJ lipoprotein	Immunogenic proteins
ACL32400.1	18991.74971	sodC	Superoxide dismutase	Immunogenic proteins
ADZ54065.1	139037.7126	vtaA4	Virulence-associated trimeric autotransporter	Immunogenic proteins
ADZ54062.1	118419.4186	vtaA1	Virulence-associated trimeric autotransporter	Immunogenic proteins
ACL31879.1	25201.37675	dsbC	Thiol:disulfide interchange protein DsbC precursor	Iron metabolism
ACL33616.1	108272.0217	hxuA	Heme/hemopexin-binding protein A	Iron metabolism
ACL32742.1	37640.63669	fbpA	Periplasmic iron-binding protein	Iron metabolism
ACL32327.1	51816.66395	HAPS_0679	Iron-sulfur cluster binding reductase	Iron metabolism
ACL32714.1	32632.15631	yfeA	Chelated iron ABC transporter	Iron metabolism; Potential virulence factor
ACL33656.1	102313.5803	tbpA	Transferrin-binding protein 1 precursor	Potential virulence factor
ACL32784.1	61260.10128	pgi	Glucose-6-phosphate isomerase	Potential virulence factor
ACL32480.1	59976.77956	manB	phosphomannomutase	Potential virulence factor
ACL33726.1	39588.34478	ompP5	Outer membrane protein P5 Precursor	Potential virulence factor
ACL33604.1	51700.50038	TolC	RND efflux system outer membrane lipoprotein	Potential virulence factor; Immunogenic proteins
ADZ54070.1	212019.0349	vtaA9	Virulence-associated trimeric autotransporter	Associated to virulence; phagocytosis resistance; Immunogenic proteins
ACL31747.1	38342.98591	lsgB	Lipopolysaccharide biosynthesis protein	Serum resistance
ACL32657.1	37332.92877	galE	UDP-glucose-4-epimerase	Serum resistance
ACL32638.1	35934.24514	HAPS_1019	ADP-heptose:LPS heptosyltransferase	Serum resistance
ACL32011.1	25577.60407	cdtA	Cytolethal distending toxin protein A	Adherence and invasion; cell cycle arrest and apoptosis; serum resistance;
ACL32010.1	30483.53774	cdtB	Cytolethal distending toxin protein B	Adherence and invasion; cell cycle arrest and apoptosis; serum resistance;
ACL32009.1	19527.029	cdtC	Cytolethal distending toxin protein C	Adherence and invasion; cell cycle arrest and apoptosis; serum resistance;

### Inflammatory Response Against Haemophilus parasuis Outer Membrane Vesicles

**Figure 4:**
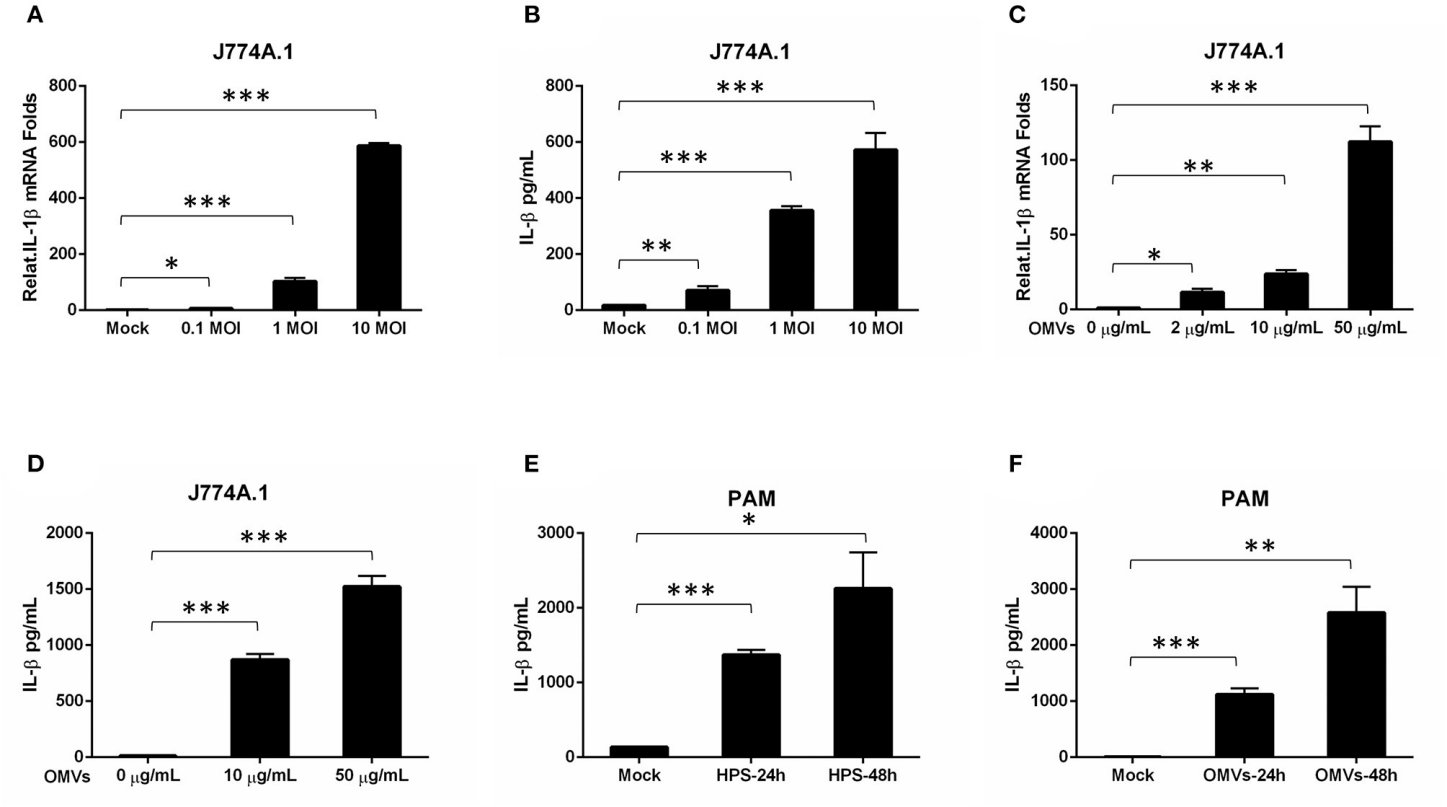
OMV is sufficient to stimulate an inflammatory response in macrophage cells. **(A,B)** Mouse Mononuclear macrophage cells (J774A.1) were either mock-infected or infected with *H. parasuis* at different doses (0.1, 1, 10 MOI) for 24 h. The mRNA levels of IL-1β in the *H. parasuis* infected cells were analyzed by qRT-PCR **(A)**. The IL-1β levels in the cell supernatants were analyzed by ELISA **(B)**. **(C)** J774A.1 were either mock-stimulated or stimulated with OMVs at 2 μg/mL, 10 μg/mL, 50 μg/mL for 24 h. The mRNA levels of IL-1β in the cell supernatants were analyzed by qRT-PCR. **(D)** J774A.1 were either mock-stimulated or stimulated with OMVs at 10 μg/mL, 50 μg/mL for 24 h. The IL-1β levels in the cell supernatants were analyzed by ELISA. **(E)** PAMs were either mock-infected or infected with *H. parasuis* at 10 MOI for different times (24 h, 48 h). The I L-1β levels in the cell supernatants were analyzed by ELISA. **(F)** PAMs were either mock-stimulated or stimulated with 10 μg/mL OMVs at different times (24 h, 48 h). The IL-1β levels in the cell supernatants were analyzed by ELISA. *p < 0.05, **p < 0.01, ***p < 0.001, Student *t* test, in comparison groups during statistical analysis.

Severe serous inflammation in multi-tissues is a typical feature of *H. parasuis* infection. To evaluate the ability of H45 infection for inducing IL-1β transcription and secretion, mouse mononuclear macrophage cells (J774A.1) were infected with H45 at different MOIs of infection. As shown in Figures 4A,B, the IL-1β messenger RNA (mRNA) expression and secretion were significantly increased in a dose-dependent manner during H45 infection. To further investigate whether OMVs take part in IL-1β secretion during *H. parasuis* H45 infection, J774A.1 were treated with OMVs at different doses for 24 h. Then, the IL-1β mRNA expression and secretion were detected by quantitative polymerase chain reaction and enzyme-linked immunosorbent assay, respectively. We found that the IL-1β expression and secretion induced by *H45*-derived OMVs are also in a dose-dependent manner (Figures 4C,D). This result suggested that OMV is an essential component in the inflammatory response caused by *H45* infection. At last, PAMs were used to confirm the results mentioned earlier. As expected, the expressed and secreted IL-1β levels were both significantly increased in PAMs after *H45* infection at 10 MOI of infection or *H45* OMVs stimulation at 10 μg/ml for 24 and 48 h (Figures 4E,F). Taken together, these results indicate that *H. parasuis H4*5 infection induces IL-1β expression and secretion, and OMVs were required for IL-1β secretion.

## Discussion

*Haemophilus parasuis* is the causative agent of Glässer's disease and also is the main bacteria in porcine mixed infection. *Haemophilus parasuis* can cause severe system inflammation alone or induce secondary infection after porcine reproductive and respiratory syndrome virus, pseudorabies virus, porcine circovirus, or another virus infection in the infected pigs (28–30). *Haemophilus parasuis* infection is increasingly harmful to the global swine industry, and the prevention and control of *H. parasuis* have always been a significant challenge, as the pathogenicity of *H. parasuis* is not fully characterized. Production of OMVs is a particular function of Gram-negative bacteria, which plays a prominent role in establishing connections between bacteria and hosts. We confirmed that *H. parasuis* secreted OMVs into the extracellular environment during *in vitro* culture and many Gram-negative bacteria. Therefore, identifying the component of *H. parasuis*-derived OMVs is a pivotal factor in ascertaining such infections' pathogenesis.

In our study, the size of extracellular vesicles of *H. parasuis* clinical isolate strain (H45) is in the range of 20–160 nm. Further proteomic analysis revealed that *H. parasuis* OMVs contain 588 proteins and are rich in cytoplasmic and membrane proteins. According to GO functional annotation analysis, a number of proteins of OMVs are involved in catalytic or binding activity functions. OMVs also carry those proteins related to transporter activity, structural molecule activity, transcription activity, molecular transducer activity, signal transducer activity, and antioxidant activity. Classification of biological processes analysis showed that the proteins of OMVs are also involved in several biological processes such as metabolic, biogenesis, ion transport and localization, and biological regulation. The characteristics mentioned earlier of OMVs indicated that OMVs might be involved in biological processes of *H. parasuis* proliferation, infection, and pathogenicity.

The trivalent inactivated vaccine of *H. parasuis* developed by our team has obtained the approval number of three new veterinary drug certificates in China. The H45 strain of *H. parasuis* is the primary strain of the vaccine. The immune protection rate of the vaccine in piglets reached 80%. Many studies have proven that native OMVs isolated from pathogenic bacteria showed strong protective efficacy against pathogen infection (31–33). In this study, we found that *H. parasuis* H45 strain-derived OMVs contain numerous immunogenic antigens, including ompP2, oppA, ompD15, palA, AfuA, LppC, espP2, and vtaA1 (Table 1). These results indicate that the OMVs of H45 have the potential to replace the whole inactivated vaccine of *H. parasuis* to protect piglets from *H. parasuis* infection. However, the immune protection efficiency of OMV in piglets still needs to be further studied.

We also confirmed the pathogenicity of *H. parasuis* OMVs by observing strong inflammatory responses in J774A.1 cells and PAMs. Furthermore, *H45*-derived OMVs stimulation also can induce the IL-1β mRNA expression and secretion in a dose- and time-dependent manner. Our data confirmed the inflammation-inducing effect of the OMVs on target cells. The characteristic of the inflammatory response induced by OMVs may be related to LPS, which is one of the most abundant host immunogenic components of OMVs. It has been demonstrated that the OMVs of enterohemorrhagic *E. coli* acted as cargo to deliver LPS into the cytosol, which trigger caspase-11-dependent effector responses *in vitro* and *in vivo* (34). OMPs and porin are major structural components of both the outer membrane and secreted vesicles involved in the formation and secretion of OMVs. The previous study has shown that loss of porin inhibited macrophage inflammatory responses to OMVs of *Klebsiella pneumoniae*, inducing extremely low levels of pro-inflammatory cytokine (35). In this study, we found that OMVs contain ompP2, which affects the adherence capacity of *H. parasuis* in PAM (11). Moreover, many virulence factors related to iron metabolism, serum resistance, adherence, and invasion were also identified. For example, vtaA9 was involved in the phagocytosis resistance during *H. parasuis* infection (8, 9); CDTs (cdtA, cdtB, and cdtC) contribute to serum resistance, adherence, and invasion in PK-15 and PUVEC (10). The hosts' inflammation response induced by OMVs might partly relate to the adherence and invasion proteins. These OMV proteins can promote the effect of pathogenic internalization. Numerous studies have clearly shown that the delivery of toxins and other virulence factors *via* OMVs essentially influences the interactions between pathogen and host cells. Hence, the functions of these virulence factors of OMVs during the *H. parasuis* infection would be further investigated.

## Conclusions

In conclusion, this study describes the typical morphology and the high-throughput proteomic characterization of OMVs derived from *H. parasuis* clinically isolated strain H45. We have identified 588 proteins in *H. parasuis*-derived OMVs and discovered several proteins associated with immunogenicity, iron metabolism, serum resistance, adherence, and invasion. Our further study shows that *H. parasuis*-derived OMVs can induce the production of pro-inflammatory cytokine IL-1β. According to these results, we speculated OMVs from *H. parasuis* were involved in the pathogenesis of this bacterium during infection.

## Materials and Methods

### Bacterial Strain, Growth Conditions, and Cell Culture

*Haemophilus parasuis* H45 strain was a clinically isolated strain in China. H45 belongs to serovar 5, which is high-level virulent and is the most popular strain in China. Bacteria were cultured in TSB (Difco Laboratories, Detroit, MI, USA) supplemented with 1 mg/ml nicotinamide adenine dinucleotide and 10% bovine serum. After incubation at 37°C overnight with circular agitation (200 rpm), bacteria were harvested at an optical density at 600 nm 0.7–0.9. A volume of each strain was mixed with 20% glycerol and stored at −80°C in cryogenic storage tubes for further use.

Mouse mononuclear macrophage cells (J774A.1) were purchased and cultured in Dulbecco's modified Eagle's medium with 10% fetal bovine serum, 100 U/ml penicillin, and 100 μg/ml streptomycin at 37°C with 5% CO_2_. Four-week-old piglets were used to isolate PAMs as previously described (36) and maintained in RPMI-1640 medium containing 10% heat-inactivated fetal bovine serum (Hyclone), 100 U/ml penicillin, 100 μg/ml streptomycin, and non-essential amino acid (Gibco) at 37°C in a humidified atmosphere of 5% CO_2._

### Purification of Outer Membrane Vesicles

The OMVs of *H. parasuis* were collected from the supernatant of bacterial culture (1,000 ml). In brief, *H. parasuis* were inoculated overnight in 5 ml of TSB. The next day, 1,000 ml of TSB with the 1 mL of starter was cultured at 37°C with shaking until the optical density at 600 nm reached 1.0. Bacterial cells were removed with centrifugation at 5,000 × *g* for 20 min at 4°C. The supernatants were concentrated with a 100-kDa hollow-fiber membrane, and then, the concentrate was vacuum filtered through a 0.22-μm membrane to remove any remaining bacteria. The OMVs were collected by ultracentrifugation at 150,000 × *g* for 4.5 h at 4°C (rotor Type 50.2 Ti, Beckman-Coulter). The pelleted OMVs were suspended with sterile phosphate-buffered saline (PBS). The resuspended OMVs were purified by density gradient centrifugation with OptiPrep [60% iodixanol (w/v)]. The OptiPrep solution was diluted with 0.85% (w/v) NaCl containing 10-mM 4-(2-hydroxyethyl) piperazine-1-ethanesulfonic acid–sodium hydroxide pH 7.4 into 45, 40, 35, 30, 25, and 20% (v/v) OptiPrep. The 45% OptiPrep solution was prepared with suspended OMVs and layered 2 ml at the bottom of an Ultra-Clear centrifuge tube (13.2 ml, Beckman Coulter). The discontinuous gradients were loaded in 2-ml increments in degressive concentrations on top of the prior layer [36]. Prepared tubes were centrifuged at 150,000 × *g* for 16 h at 4°C (rotor SW 41 Ti, BeckmanCoulter). Each 1-ml fraction was carefully collected by pipet from top to bottom. The OMV content of each fraction was analyzed by 12% SDS-PAGE. The fractions containing OMVs were collected and pooled into an ultracentrifugation tube with 27-ml sterile PBS (at least 10-fold of the sample volume) and then ultracentrifuged at 200,500 × *g* for 2 h at 4°C (rotor Type 45 Ti, Beckman Coulter) to remove the OptiPrep solution. The purified OMVs were resuspended with sterile PBS, and the aliquots were stored at −80°C for future use.

### Transmission Electron Microscopy

Transmission electron microscopy was used to investigate OMV morphology. A drop of OMV suspension was placed on Formvar/carbon-coated grids and adsorbed for 5 min. Grids were washed with distilled water and blotted with filter paper. For negative staining, grids were treated with 1.5% (wt/vol) uranyl acetate for 1 min, air-dried, and viewed with a Hitachi HT7700 electron microscope operating at 80 kV.

### Sodium Dodecyl Sulfate Poly-Acrylamide Gel Electrophoresis

OMV proteins were quantified by BCA Protein Assay Kit (Thermo fisher scientific, Cat# 23225). Vesicles proteins from *H. parasuis* H45 (Serum type 5) were analyzed by 12% SDS-PAGE. The gel was stained with Coomassie brilliant blue. Protein molecular weight standard (size range 35–180 KDa, Thermo Fisher Scientific) was used.

### Liquid Chromatography With Tandem Mass Spectrometry (Mass Spectrometry of Outer Membrane Vesicles)

The vesicle proteins were separated by SDS-PAGE and digested with the trypsin enzyme. The digested peptide liquid was desalted and freeze-dried. The dried peptide samples were reconstituted with mobile phase A (2% ACN, 0.1% FA), and the supernatant after centrifugation at 20,000 × *g* for 10 min was taken for high-performance liquid chromatography–ESI-MS/MS analysis. The peptide samples were subjected to Shimadzu LC-20AD model nanoliter liquid chromatography (75 μm × 3 μm × 15 cm, C18 column) at a flow rate of 300 nl/min. The liquid phase chromatography-separated peptides were passed to an ESI-MS/MS, which was carried out using a TripleTOF 5600 (SCIEX, Framingham, MA, USA) mass spectrometer equipped with a Nanospray III source, and coupled with an emitter which drawn from quartz material (New Objectives, Woburn, MA, USA). For data acquisition, the mass spectrometer parameters were set as follows: ion source spray voltage 2,300 V, nitrogen pressure 35 psi, spray gas 15, and spray interface temperature 150°C. Scanning in high sensitivity mode, the MS1 scan cumulative time was 250 ms, and the scan quality range was 350–1,500 Da. Based on the MS1 scanning information, according to the ionic strength in the MS1 spectrum from high to low, the first 30 ions exceeding 150 cp were selected for fragmentation, and the MS2 information was scanned. The scan accumulation time of the MS2 mass spectrum was 50 ms. The collision energy was set to “rolling collision energy.”

### Bioinformatics Protein Identification

The protein identification uses experimental MS/MS data and aligns them with theoretical MS/MS data from the database to obtain results. The process starts from converting raw MS data into a peak list and then searching matches in the Mascot search engine (Mascot 2.3.02). The UniProt protein database and National Center for Biotechnology Information database were selected. We used *e*-value ≤ 0.05 from Mascot as a filtering condition in this pipeline (the PSM FDR column is the Mascot value). Then, based on the parsimony principle, we performed protein inference on peptides and generated a series of protein groups. Finally, functional annotation analyses such as GO, Clusters of Orthologous Groups of protein/EuKaryotic Orthologous Groups, and Pathway analysis are performed from the final protein identification list.

### Enzyme-Linked Immunosorbent Assay

J774A.1 or PAMs cells were stimulated by *H. parasuis* or OMVs as indicated. After 24 or 48 h, supernatants from cell culture were harvested and assayed with the production of murine IL-1β (R&D Systems, Cat# MLB00C) and swine IL-1β (RayBio, Cat# ELP-IL1b-1) by enzyme-linked immunosorbent assay, in accordance with the manufacturer's instructions. The absorbance was quantified at 450 nm by a microplate spectrophotometer (Synergy H1, BioTek, USA).

## Data Availability Statement

The original contributions of mass spectrometry proteomics data presented in the study are publicly available. This data can be found here: http://proteomecentral.proteomexchange.org. Further inquiries can be directed to the corresponding author.

## Author Contributions

KZ and CL designed and supervised the research. KZ wrote the paper and revised the manuscript. KZ, PC, ZB, DY, SS, YL, ZJ, HG, PC, and RC performed and analyzed the experiments. All authors contributed to the article and approved the submitted version.

## Funding

This study was supported by the National Natural Science Foundation of China (32002298), Independent Research and Development Projects of Maoming Laboratory (2021ZZ003), the Science and Technology Planning Project of Guangzhou (202102020385), and the Special Fund for Scientific Innovation Strategy—Construction of High-Level Academy of Agriculture Science (R2019YJ-YB2005, R2020QD-047) and the Special fund for scientific innovation strategy-construction of high level Academy of Agriculture Science-Prominent Talents (R2020PYJC001).

## Conflict of Interest

The authors declare that the research was conducted in the absence of any commercial or financial relationships that could be construed as a potential conflict of interest.

## Publisher's Note

All claims expressed in this article are solely those of the authors and do not necessarily represent those of their affiliated organizations, or those of the publisher, the editors and the reviewers. Any product that may be evaluated in this article, or claim that may be made by its manufacturer, is not guaranteed or endorsed by the publisher.

## References

[1] Moller K Kilian M. V factor-dependent members of the family Pasteurellaceae in the porcine upper respiratory tract. J Clin Microbiol. (1990) 28:2711–6. 10.1128/jcm.28.12.2711-2716.1990 2280002 PMC268260

[2] Rafiee M Blackall PJ. Establishment, validation and use of the Kielstein-Rapp-Gabrielson serotyping scheme for *Haemophilus parasuis*. Aust Vet J. (2000) 78:172–4. 10.1111/j.1751-0813.2000.tb10586.x 10860155

[3] Del Rio ML Gutierrez CB Rodriguez Ferri EF. Value of indirect hemagglutination and coagglutination tests for serotyping *Haemophilus parasuis*. J Clin Microbiol. (2003) 41:880–2. 10.1128/JCM.41.2.880-882.2003 12574306 PMC149707

[4] Cai X Chen H Blackall PJ Yin Z Wang L Liu Z. Serological characterization of *Haemophilus parasuis* isolates from China. Vet Microbiol. (2005) 111:231–6. 10.1016/j.vetmic.2005.07.007 16271834

[5] Zhang J Xu C Guo L Ke B Ke C Zhang B. A rapid pulsed-field gel electrophoresis method of genotyping *Haemophilus parasuis* isolates. Lett Appl Microbiol. (2011) 52:589–95. 10.1111/j.1472-765X.2011.03048.x 21507027

[6] Zhao Y Wang Q Li J Lin X Huang X Fang B. Epidemiology of *Haemophilus parasuis* isolates from pigs in China using serotyping, antimicrobial susceptibility, biofilm formation and ERIC-PCR genotyping. PeerJ. (2018) 6:e5040. 10.7717/peerj.5040 29915708 PMC6004116

[7] Bouchet B Vanier G Jacques M Auger E Gottschalk M. Studies on the interactions of *Haemophilus parasuis* with porcine epithelial tracheal cells: limited role of LOS in apoptosis and pro-inflammatory cytokine release. Microb Pathog. (2009) 46:108–13. 10.1016/j.micpath.2008.10.008 19013513

[8] Olvera A Ballester M Nofrarias M Sibila M Aragon V. Differences in phagocytosis susceptibility in *Haemophilus parasuis* strains. Vet Res. (2009) 40:24. 10.1051/vetres/2009007 19239855 PMC2695031

[9] Costa-Hurtado M Ballester M Galofre-Mila N Darji A Aragon V. VtaA8 and VtaA9 from *Haemophilus parasuis* delay phagocytosis by alveolar macrophages. Vet Res. (2012) 43:57. 10.1186/1297-9716-43-57 22839779 PMC3462726

[10] Zhang B He Y Xu C Xu L Feng S Liao M. Cytolethal distending toxin (CDT) of the *Haemophilus parasuis* SC096 strain contributes to serum resistance and adherence to and invasion of PK-15 and PUVEC cells. Vet Microbiol. (2012) 157:237–42. 10.1016/j.vetmic.2011.12.002 22221379

[11] Zhang B Xu C Liao M. Outer membrane protein P2 of the *Haemophilus parasuis* SC096 strain contributes to adherence to porcine alveolar macrophages cells. Vet Microbiol. (2012) 158:226–7. 10.1016/j.vetmic.2012.01.023 22342495

[12] Xu C Zhang L Zhang B Feng S Zhou S Li J. Involvement of lipooligosaccharide heptose residues of *Haemophilus parasuis* SC096 strain in serum resistance, adhesion and invasion. Vet J. (2013) 195:200–4. 10.1016/j.tvjl.2012.06.017 22857892

[13] Costa-Hurtado M Garcia-Rodriguez L Lopez-Serrano S Aragon V. *Haemophilus parasuis* VtaA2 is involved in adhesion to extracellular proteins. Vet Res. (2019) 50:69. 10.1186/s13567-019-0687-2 31547880 PMC6755704

[14] Beveridge TJ. Structures of gram-negative cell walls and their derived membrane vesicles. J Bacteriol. (1999) 181:4725–33. 10.1128/JB.181.16.4725-4733.1999 10438737 PMC93954

[15] Jan AT. Outer membrane vesicles (OMVs) of gram-negative bacteria: a perspective update. Front Microbiol. (2017) 8:1053. 10.3389/fmicb.2017.01053 28649237 PMC5465292

[16] McBroom AJ Kuehn MJ. Release of outer membrane vesicles by Gram-negative bacteria is a novel envelope stress response. Mol Microbiol. (2007) 63:545–58. 10.1111/j.1365-2006.05522.x 17163978 PMC1868505

[17] Devoe IW Gilchrist JE. Release of endotoxin in the form of cell wall blebs during *in vitro* growth of *Neisseria meningitidis*. J Exp Med. (1973) 138:1156–67. 10.1084/jem.138.5.1156 4200775 PMC2139435

[18] Kadurugamuwa JL Beveridge TJ. Delivery of the non-membrane-permeative antibiotic gentamicin into mammalian cells by using Shigella flexneri membrane vesicles. Antimicrob Agents Chemother. (1998) 42:1476–83. 10.1128/AAC.42.6.1476 9624497 PMC105625

[19] Keenan J Day T Neal S Cook B Perez-Perez G Allardyce R. A role for the bacterial outer membrane in the pathogenesis of Helicobacter pylori infection. FEMS Microbiol Lett. (2000) 182:259–64. 10.1111/j.1574-6968.2000.tb08905.x 10620676

[20] Kadurugamuwa JL Beveridge TJ. Virulence factors are released from Pseudomonas aeruginosa in association with membrane vesicles during normal growth and exposure to gentamicin: a novel mechanism of enzyme secretion. J Bacteriol. (1995) 177:3998–4008. 10.1128/jb.177.14.3998-4008.1995 7608073 PMC177130

[21] Ellis TN Kuehn MJ. Virulence and immunomodulatory roles of bacterial outer membrane vesicles. Microbiol Mol Biol Rev. (2010) 74:81–94. 10.1128/MMBR.00031-09 20197500 PMC2832350

[22] Mashburn LM Whiteley M. Membrane vesicles traffic signals and facilitate group activities in a prokaryote. Nature. (2005) 437:422–5. 10.1038/nature03925 16163359

[23] Toyofuku M Morinaga K Hashimoto Y Uhl J Shimamura H Inaba H. Membrane vesicle-mediated bacterial communication. ISME J. (2017) 11:1504–9. 10.1038/ismej.2017.13 28282039 PMC5437348

[24] Brameyer S Plener L Muller A Klingl A Wanner G Jung K. Outer membrane vesicles facilitate trafficking of the hydrophobic signaling molecule CAI-1 between *Vibrio harveyi* cells. J Bacteriol. (2018) 200:e00740–17. 10.1128/JB.00740-17 29555694 PMC6040191

[25] Roier S Zingl FG Cakar F Durakovic S Kohl P Eichmann TO. A novel mechanism for the biogenesis of outer membrane vesicles in gram-negative bacteria. Nat Commun. (2016) 7:10515. 10.1038/ncomms10515 26806181 PMC4737802

[26] Zingl FG Kohl P Cakar F Leitner DR Mitterer F Bonnington KE. Outer membrane vesiculation facilitates surface exchange and *in vivo* adaptation of vibrio cholerae. Cell Host Microbe. (2020) 27:225–37.e228. 10.1016/j.chom.2019.12.002 31901519 PMC7155939

[27] Parrow NL Fleming RE Minnick MF. Sequestration and scavenging of iron in infection. Infect Immun. (2013) 81:3503–14. 10.1128/IAI.00602-13 23836822 PMC3811770

[28] Palzer A Ritzmann M Wolf G Heinritzi K. Associations between pathogens in healthy pigs and pigs with pneumonia. Vet Rec. (2008) 162:267–71. 10.1136/vr.162.9.267 18310558

[29] Zhao Z Qin Y Lai Z Peng L Cai X Wang L. Microbial ecology of swine farms and PRRS vaccine vaccination strategies. Vet Microbiol. (2012) 155:247–56. 10.1016/j.vetmic.2011.09.028 22014373

[30] Palzer A Eddicks M Zoels S Stark J Reese S Strutzberg-Minder K. Field evaluation of the efficacy, compatibility and serologic profiling of a combined vaccine against porcine reproductive and respiratory syndrome and *Haemophilus parasuis* in nursery pigs. Prev Vet Med. (2015) 119:134–40. 10.1016/j.prevetmed.2015.03.005 25819628

[31] Liu Q Yi J Liang K Zhang X Liu Q. Salmonella choleraesuis outer membrane vesicles: proteomics and immunogenicity. J Basic Microbiol. (2017) 57:852–61. 10.1002/jobm.201700153 28745825

[32] Hays MP Houben D Yang Y Luirink J Hardwidge PR. Immunization with skp delivered on outer membrane vesicles protects mice against enterotoxigenic escherichia coli challenge. Front Cell Infect Microbiol. (2018) 8:132. 10.3389/fcimb.2018.00132 29765911 PMC5938412

[33] Solanki KS Varshney R Qureshi S Thomas P Singh R Agrawal A. Non-infectious outer membrane vesicles derived from Brucella abortus S19Deltaper as an alternative acellular vaccine protects mice against virulent challenge. Int Immunopharmacol. (2021) 90:107148. 10.1016/j.intimp.2020.107148 33189614

[34] Vanaja SK Russo AJ Behl B Banerjee I Yankova M Deshmukh SD. Bacterial outer membrane vesicles mediate cytosolic localization of LPS and caspase-11 activation. Cell. (2016) 165:1106–19. 10.1016/j.cell.2016.04.015 27156449 PMC4874922

[35] Turner KL Cahill BK Dilello SK Gutel D Brunson DN Alberti S. Porin Loss Impacts the Host Inflammatory Response to Outer Membrane Vesicles of Klebsiella pneumoniae. Antimicrob Agents Chemother. (2015) 60:1360–9. 10.1128/AAC.01627-15 26666932 PMC4776010

[36] Zhang H Guo X Ge X Chen Y Sun Q Yang H. Changes in the cellular proteins of pulmonary alveolar macrophage infected with porcine reproductive and respiratory syndrome virus by proteomics analysis. J. Proteome Res. (2009) 8:3091–7. 10.1021/pr900002f 19341299

